# Cl- and Al-Doped Argyrodite Solid Electrolyte Li_6_PS_5_Cl for All-Solid-State Lithium Batteries with Improved Ionic Conductivity

**DOI:** 10.3390/nano12244355

**Published:** 2022-12-07

**Authors:** Yeong Jun Choi, Sun-I Kim, Mingyu Son, Jung Woo Lee, Duck Hyun Lee

**Affiliations:** 1 Green Materials and Processes R&D Group, Korea Institute of Industrial Technology, Ulsan 44413, Republic of Korea; 2 Department of Materials Science and Engineering, Pusan National University, Busan 46241, Republic of Korea; 3 School of Materials Science and Engineering, Andong National University, Andong 36729, Republic of Korea

**Keywords:** all-solid-state battery, solid electrolyte, argyrodite, ionic conductivity, doping

## Abstract

Argyrodite solid electrolytes such as lithium phosphorus sulfur chloride (Li_6_PS_5_Cl) have recently attracted great attention due to their excellent lithium-ion transport properties, which are applicable to all-solid-state lithium batteries. In this study, we report the improved ionic conductivity of an argyrodite solid electrolyte, Li_6_PS_5_Cl, in all-solid-state lithium batteries via the co-doping of chlorine (Cl) and aluminum (Al) elements. Electrochemical analysis was conducted on the doped argyrodite structure of Li_6_PS_5_Cl, which revealed that the substitution of cations and anions greatly improved the ionic conductivity of solid electrolytes. The ionic conductivity of the Cl- and Al-doped Li_6_PS_5_Cl (Li_5.4_Al_0.1_PS_4.7_Cl_1.3_) electrolyte was 7.29 × 10^−3^ S cm^−1^ at room temperature, which is 4.7 times higher than that of Li_6_PS_5_Cl. The Arrhenius plot of the Li_5.4_Al_0.1_PS_4.7_Cl_1.3_ electrolyte further elucidated its low activation energy at 0.09 eV.

## 1. Introduction

Lithium-ion batteries are the core technology for electric vehicles and energy storage systems due to their high energy density and long cycle life [1,2,3,4]. However, most commercialized lithium-ion batteries use liquid electrolytes, which increases the risk of ignition and explosion [5,6,7,8]. All-solid-state batteries use solid electrolytes, which are safer than flammable organic solvents, and they have a high energy density as well. Thus, they are expected to outperform lithium-ion batteries in most aspects [9,10,11,12]. Despite these advantages, all-solid-state batteries have faced challenges in commercialization because the ionic conductivity of the solid electrolyte is generally several tens of times lower than that of the liquid electrolyte [7,13]. Since ionic conductivity is linked to cell performance, high ionic conductivity is required to realize high charge/discharge rates and capacity [5]. Therefore, extensive research has been conducted to improve the ionic conductivity of solid electrolytes [14].

Solid electrolytes are divided into organic solid electrolytes (polymer-based) and inorganic solid electrolytes (oxide-based or sulfide-based) depending on the material used. The polymer-based solid electrolyte has excellent mechanical properties and is easy to apply as a flexible battery but has very low ionic conductivity (10^−5^–10^−8^ S cm^−1^) [15]. In contrast, oxide-based solid electrolytes have high voltage stability and are stable in air but require high temperatures (800–1000 °C) for the sintering process and have low ionic conductivities (10^−5^–10^−6^ S cm^−1^) [16]. Among many solid electrolytes, sulfide-based solid electrolytes, particularly argyrodite solid electrolytes, having the structure of lithium phosphorus sulfur X (Li_6_PS_5_X, where X = Cl, Br, or I), are attracting attention because they have relatively high ionic conductivities and enable the synthesis of materials with various compositions through the substitution of cations and anions [17].

Li_6_PS_5_Cl has a relatively high ionic conductivity of 10^−3^ S cm^−1^ as compared to Li_6_PS_5_I and Li_6_PS_5_Br, which have low ionic conductivities in the range of 10^−6^–10^−4^ S cm^−1^ [18,19,20]. Therefore, the Li_6_PS_5_Cl solid electrolyte has received great attention for its potential use in commercial all-solid-state batteries. Generally, argyrodite Li_6_PS_5_Cl is synthesized by ball milling precursors and annealing [21,22]. Yu et al. prepared a Li_6_PS_5_Cl solid electrolyte with a conductivity of 1.1 × 10^−3^ S cm^−1^ by ball milling the precursors at 550 rpm for 10 h and annealing at 550 °C for 5 h [23]. Recently, several studies have reported the increased ionic conductivity of solid electrolytes with ionic doping [17,24,25,26,27,28,29]. Doping is the intentional introduction of impurities into an intrinsic material to modulate its electrical, optical, and structural properties. Zhang et al. reported Al^3+^/B^3+^ as a partial substitution of lithium (Li) sites for Li_6_PS_5_X (X = Cl and Br) based on X-ray diffraction (XRD) refinement, and Li_5.4_Al_0.2_PS_5_Br showed an increased ionic conductivity of 2.4 × 10^−3^ S cm^−1^ at room temperature [24]. In another group, tellurium (Te)-doped Li_6.25_PTe_0.125_S_5.125_Cl_0.75_ exhibited a relatively high ionic conductivity of 4.5 × 10^−3^ S cm^−1^ at room temperature [29].

In this study, we report the co-doping of cations and halogen elements into argyrodite LI_6_PS_5_Cl to improve the ionic conductivity of the solid electrolyte for all-solid-state lithium batteries. The crystal structure of cubic Li_6_PS_5_Cl within the argyrodite group consists of PS_4_^3−^ tetrahedra and isolated S^2−^/Cl^−^ and Li^+^ ions [20]. Li^+^ sites are capable of various Li^+^ ion jumps. The high ionic conductivity is indicated by disordered phases of S^2−^ and Cl^−^ at two crystallographic positions (4a, 4c Wyckoff) in the cubic lattice. The substitution of Cl^−^ in the lattice of two mixed S^2−^/Cl^−^ sites induces vacancies at Li^+^ sites [30]. In addition, the substitution of cations with a radius similar to that of Li^+^ ions increases the vacancy concentration of Li^+^ sites [24]. The formation of the vacancy site provides a diffusion pathway to transport Li ions. The substitution of Al^3+^ and Cl^−^ was induced to increase the vacancy sites for Li^+^. The effect of Cl and Al doping was observed with XRD, Raman analysis, and X-ray photoelectron spectroscopy (XPS), and the electrochemical properties of the doped solid electrolytes were measured with the Nyquist plot and Arrhenius plot. 

## 2. Materials and Methods

### 2.1. Synthesis of Solid Electrolytes

Li_6−x−3y_Al_y_PS_5−x_Cl_1+x_ with different doping amounts was synthesized with appropriate stoichiometric ratios of lithium sulfide (Li_2_S, 99.98%, Sigma Aldrich, St. Louis, MO, USA), phosphorus pentasulfide (P_2_S_5_, 99%, Sigma Aldrich, St. Louis, MO, USA), lithium chloride (LiCl, 99.98%, Sigma Aldrich, St. Louis, MO, USA), and aluminum sulfide (Al_2_S_3_, 98%, Sigma Aldrich, St. Louis, MO, USA). The precursors and a zirconia ball (diameter 10 mm) were placed in high-density polyethylene (HDPE), and then mechanical milling was performed at 550 rpm for 5 h using a swing planetary mixer (HSPM-V1.5, Hantech Co., Ltd., Gunpo-si, Republic of Korea). The milling process was performed in such a way that a 5 min break time was given after every 30 min of milling. The mixture obtained after ball milling was sealed in a quartz tube, and annealing was performed at 550 °C (3.5 °C/min) in a tube furnace (Na-AF15, Nasiltech Co., Ltd., Seoul, Republic of Korea) for 10 h. Since the sulfide-based solid electrolyte was decomposed while generating H_2_S gas in the reaction with moisture in the atmosphere, all synthesis processes were performed in a glove box, which had an inactive argon (Ar) atmosphere.

### 2.2. Material Characterization

XRD measurements of the Li_6−x−3y_Al_y_PS_5−x_Cl_1+x_ electrolytes were performed with an X-ray diffractometer (Rigaku D/MAX-2500V/PC, Tokyo, Japan). Cu Kα (wavelength = 1.5406 Å) was used for the X-ray, and the polyimide film was sealed in the glove box to maintain an Ar environment during the analysis process due to the high reactivity of the sulfide-based solid electrolyte with moisture in the atmosphere. The scan rate was 2°/min, and the 2θ range was measured from 10° to 70°. Rietveld refinements of XRD data were conducted using GSAS II software. Raman spectrum measurements were performed using confocal Raman (Alpha300R, WITec, Ulm, Germany) analysis equipment using a laser wavelength of 532 nm. FE-SEM measurements were performed using (SU8020, Hitachi, Tokyo, Japan). To observe the effect of particle size and distribution in more detail, the particle size and shape of the electrolyte were observed after uniform dispersion using a spin coater (NSF-150DP, Rhardos, Co., Ltd., Seoul, Republic of Korea). XPS was measured using the (Multilab-2000, Thermo Fisher Scientific Inc., Waltham, MA, USA). The chemical state of the element was investigated through the X-ray source of the twin anode (Al Kα, hν = 1486.6 eV) and monochromatic guns.

### 2.3. Measurement of Electrochemical Performance

To calculate the ionic conductivity of the Li_6−x−3y_Al_y_PS_5−x_Cl_1+x_ electrolytes, the solid electrolyte powder was prepared into a circular pellet with a diameter of 10 mm and 0.5–0.7 mm thickness by uniaxial pressure molding at 15 MPa. An ion-blocking cell was manufactured by pressing stainless steel (SUS), which is an ion-blocking electrode, on both sides of the solid electrolyte. Electrochemical impedance spectroscopy (EIS) was performed using an electrochemical workstation (SP-300 Potentiostat, BioLogic, Seyssinet-Pariset, France). It was measured in a frequency range of 1 Hz to 7 MHz with an amplitude of 50 mV. The ionic conductivity was calculated by the equation σ = *L/RS*, where *L* is the thickness of the electrolyte, *S* is the area of the electrolyte, and *R* is the resistance of the measured solid electrolyte. The activation energy (E_a_) was calculated as the slope of the Arrhenius plot in the temperature range of 25–100 °C. The cell manufacturing process was carried out in a glove box in an inactive Ar atmosphere.

## 3. Results and Discussion

### 3.1. Cl-Doped Solid Electrolyte

**Figure 1:**
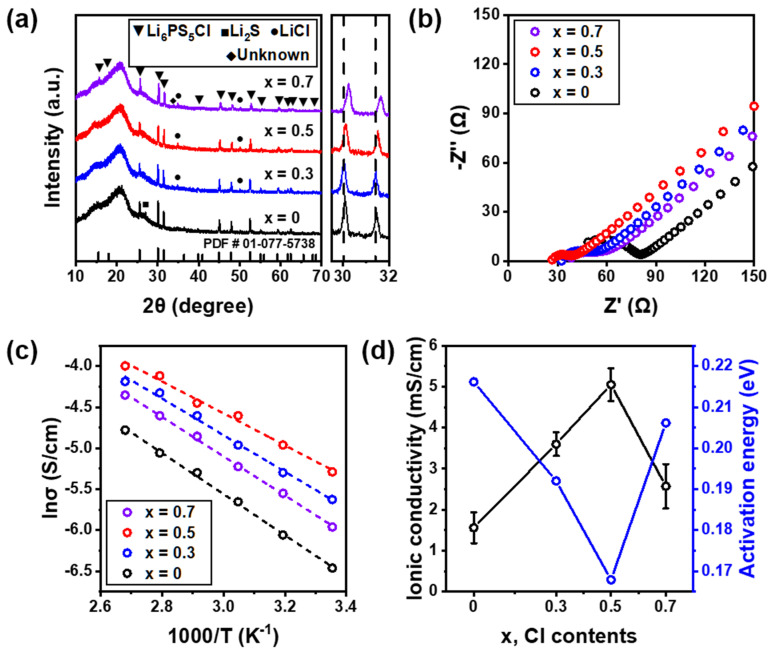
(**a**) XRD patterns of Li_6−x_PS_5−x_Cl_1+x_ (0 ≤ x ≤ 0.7) electrolytes. (**b**) Nyquist plots of Li_6−x_PS_5−x_Cl_1+x_ (0 ≤ x ≤ 0.7) electrolytes measured at 25 °C. (**c**) Arrhenius plots of Li_6−x_PS_5−x_Cl_1+x_ (0 ≤ x ≤ 0.7) electrolytes measured in the range of 25 to 100 °C. (**d**) Activation energy (E_a_) and ionic conductivity of Li_6−x_PS_5−x_Cl_1+x_ (0 ≤ x ≤ 0.7) electrolytes as a function of the Cl content.

The solid Li_6_PS_5_Cl electrolyte with Cl substitution was prepared by high-energy ball-milling and annealing processes, and Figure 1a shows the measured XRD patterns of Li_6−x_PS_5−x_Cl_1+x_ (0 ≤ x ≤ 0.7) electrolytes. As a clear phase change was not observed in any of the Cl-doped electrolytes, it was confirmed that they had an argyrodite *F*$\bar{43}$*m* structure of Li_6_PS_5_Cl (PDF # 01-077-5738). A slight Li_2_S peak was observed for Li_6_PS_5_Cl; however, the peak disappeared, and residual LiCl was observed with an increase in Cl content. An unknown phase was also observed in higher-Cl-content electrolytes (x = 0.7), and it represents the solubility limit of Cl in Li_6−x_PS_5−x_Cl_1+x_ at x = 0.7. The argyrodite diffraction peak at 30–32° showed a slight shift to a higher angle as the Cl content increased. This indicates a decrease in the crystal lattice parameters due to Cl doping at the S^2−^ site because the ionic radius of Cl^−^ is smaller than that of the S^2−^ site. The halo pattern at low angles (10–20°) was caused by the polyimide film used to block air [31].

The Nyquist plots of Li_6−x_PS_5−x_Cl_1+x_ (0 ≤ x ≤ 0.7) electrolytes measured at 25 °C are shown in Figure 1b. The impedance of the electrolyte gradually decreased with the increase in Cl content up to x = 0.5, and thereafter, it increased with a further increase in Cl content. The Arrhenius plots obtained by heating the electrolyte from 25 to 100 °C in 15 °C steps are shown in Figure 1c, and the E_a_ and ionic conductivity of prepared electrolytes are shown in Figure 1d. E_a_ was calculated with the slope of the linear Arrhenius plots according to the Arrhenius equation, σ = *A* exp(−E_a_/*kT*), where *T* is the absolute temperature, *A* is a pre-exponential factor, and *k* is the Boltzmann constant. The E_a_ value of the Li_6_PS_5_Cl electrolyte was 0.22 eV, and the E_a_ values of x = 0.3, 0.5, and 0.7 electrolytes were 0.18, 0.17, and 0.21 eV, respectively. The x = 0.5 electrolyte had the lowest E_a_. As the Cl content increased, the ionic conductivity increased, reached a maximum at x = 0.5, and then decreased. The Li_5.5_PS_4.5_Cl_1.5_ electrolyte obtained an ionic conductivity of 5.05 × 10^−3^ S cm^−1^ and an E_a_ of 0.17 eV, and the ionic conductivity was 3.2 times higher than that of Li_6_PS_5_Cl. This indicates that Cl doping can reduce E_a_ while improving the ionic conductivity. The substitution of the halogen element Cl in the Li_6_PS_5_Cl structure leads to the increased disorder of the mixed Cl^−^/S^2−^ sites at the two crystallographic positions (4a, 4c Wyckoff) of the cubic lattice, causing vacancies of Li^+^ sites [30]. Site disorder occurs because Cl^−^ ions share two sites (4a and 4c) with S^2−^, which changes the energy of Li^+^ ion diffusion [32]. The higher the Cl^−^/S^2−^ ratio, the greater the proportion of Li vacancies compared to Li_6_PS_5_Cl. Therefore, Cl doping causes the disorder of the Cl^−^/S^2−^ site, and the ionic conductivity can be improved by forming a vacancy site in Li due to the increase in the disorder of the Cl^−^/S^2−^ site. Attempts to introduce additional halogen elements into the Li_5.5_PS_4.5_Cl_1.5_ structure result in the significant solvation of LiCl. The solvation limit can be determined by the thermodynamic instability of the lattice at a high vacancy content. As can be seen from the XRD pattern shown in Figure 1a, an unknown image is formed at x = 0.7. This eventually saturates, indicating that there is a limit to replacing the S^2−^ site with Cl^−^.

### 3.2. Al-Doped Solid Electrolyte

**Figure 2:**
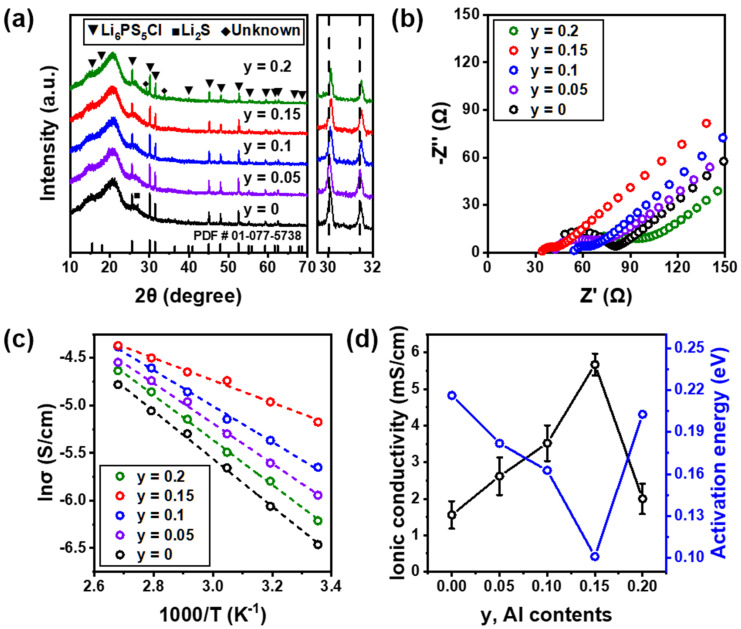
(**a**) XRD patterns of Li_6−3y_Al_y_PS_5_Cl (0 ≤ y ≤ 0.2) electrolytes. (**b**) Nyquist plots of Li_6−3y_Al_y_PS_5_Cl (0 ≤ y ≤ 0.2) electrolytes measured at 25 °C. (**c**) Arrhenius plots of Li_6−3y_Al_y_PS_5_Cl (0 ≤ y ≤ 0.2) electrolytes measured in the range of 25 to 100 °C. (**d**) E_a_ and ionic conductivity of Li_6−3y_Al_y_PS_5_Cl (0 ≤ y ≤ 0.2) electrolytes as a function of the Al content.

The solid electrolyte with the cation element substituted with Al was prepared by high-energy ball-milling and annealing processes, and the measured XRD patterns of Li_6−3y_Al_y_PS_5_Cl (0 ≤ y ≤ 0.2) electrolytes are shown in Figure 2a. None of the Al-doped electrolytes showed a clear phase change, and it was confirmed that they had an argyrodite *F*$\bar{43}$*m* structure of Li_6_PS_5_Cl (PDF # 01-077-5738). A slight Li_2_S peak disappeared with the increase in Al content, and an unknown phase was observed at a higher Al content in electrolytes (y = 0.2). This means that the solubility limit was at y = 0.2. In addition, the argyrodite diffraction peak at 30–32° showed a slight shift to a higher angle with increased Al content. This indicates a decrease in the crystal lattice parameters due to Al^3+^ doping at the Li^+^ site because the ionic radius of Al^3+^ is smaller than that of the Li^+^ site. Figure 2b shows Nyquist plots of Li_6−3y_Al_y_PS_5_Cl (0 ≤ y ≤ 0.2) electrolytes measured at 25 °C. As the Al content increased, the impedance of the electrolyte gradually decreased, and when y = 0.2, the impedance increased. The Arrhenius plots obtained by heating the electrolyte from 25 to 100 °C in 15 °C steps are shown in Figure 2c. E_a_ and ionic conductivity are shown in Figure 2d. The E_a_ values at y = 0.05, 0.1, 0.15, and 0.2 electrolytes were 0.18, 0.16, 0.10, and 0.20 eV, respectively. The Li_6−3y_Al_y_PS_5_Cl electrolyte with y = 0.15 had the lowest E_a_. As the Al content increased, the ionic conductivity increased, reached a maximum at y = 0.15, and then decreased. The Li_5.55_Al_0.15_PS_5_Cl electrolyte obtained an ionic conductivity of 5.67 × 10^−3^ S cm^−1^ and an E_a_ of 0.10 eV, and the ionic conductivity increased by 3.6 times as compared to Li_6_PS_5_Cl. This indicates that Al doping can simultaneously improve the ionic conductivity while decreasing E_a_. For ion transport in solid electrolytes, vacancies created by ionic point defects form diffusion pathways that can transport Li^+^ ions [24]. Therefore, the vacancy concentration of Li^+^ sites is increased by substituting a cation with a radius similar to that of Li^+^ ions. This means that the ionic conductivity was improved by forming a diffusion pathway. As can be seen from the XRD pattern shown in Figure 2a, the decrease in the ionic conductivity of the electrolyte at y = 0.2 is considered to have contributed to the decrease in the ionic conductivity due to the formation of an unknown phase.

### 3.3. Structural Analysis of Co-Doped Solid Electrolyte

**Figure 3:**
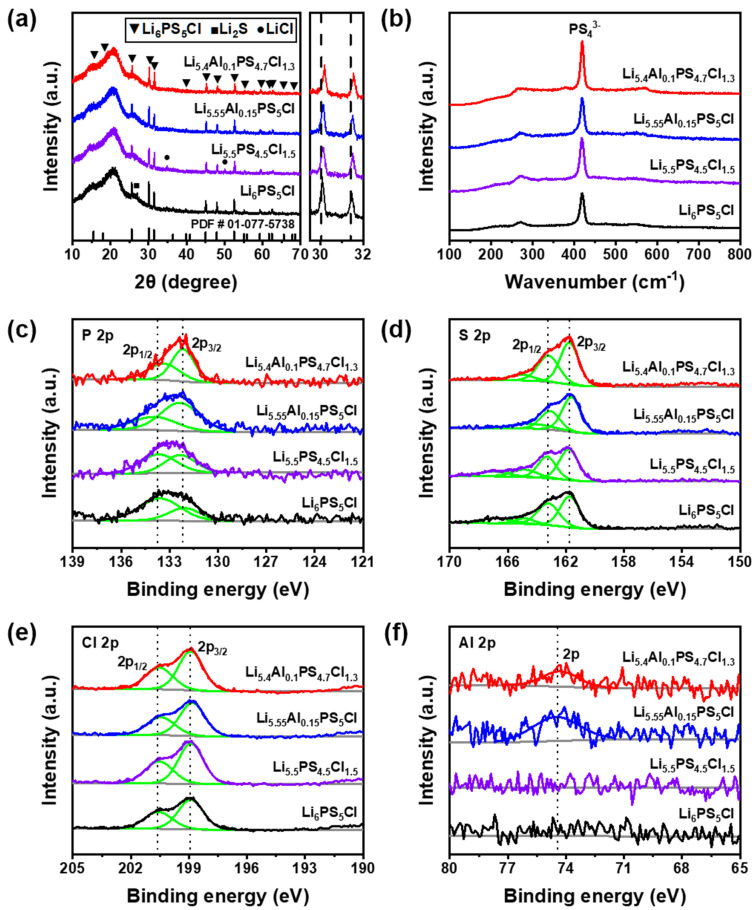
(**a**) XRD patterns of Li_6_PS_5_Cl, Li_5.5_PS_4.5_Cl_1.5_, Li_5.55_Al_0.15_PS_5_Cl, and Li_5.4_Al_0.1_PS_4.7_Cl_1.3_ electrolytes. (**b**) Raman spectra of Li_6_PS_5_Cl, Li_5.5_PS_4.5_Cl_1.5_, Li_5.55_Al_0.15_PS_5_Cl, and Li_5.4_Al_0.1_PS_4.7_Cl_1.3_ electrolytes. XPS spectra of Li_6_PS_5_Cl, Li_5.5_PS_4.5_Cl_1.5_, Li_5.55_Al_0.15_PS_5_Cl, and Li_5.4_Al_0.1_PS_4.7_Cl_1.3_ electrolytes for (**c**) P 2p, (**d**) S 2p, (**e**) Cl 2p, and (**f**) Al 2p.

To further increase the ionic conductivity of Li_6−x−3y_Al_y_PS_5−x_Cl_1+x_ (0 ≤ x ≤ 0.7, 0 ≤ y ≤ 0.2) solid electrolytes, they were co-doped with Cl and Al elements, and the ionic conductivity was measured at 25 °C. The morphology of the Li_5.4_Al_0.1_PS_4.7_Cl_1.3_ electrolyte obtained by field-emission scanning electron microscopy (FE-SEM) shows that the particle size of the electrolyte ranges from 0.5 to 1 μm (Figure S1). Figure 3a shows the measured XRD patterns of the prepared electrolytes (x = 0, y = 0; x = 0.5, y = 0; x = 0, y = 0.15; and x = 0.3, y = 0.1). It was confirmed that the Al and Cl co-doped (x = 0.3, y = 0.1) electrolyte did not show a clear phase change and had an argyrodite structure of Li_6_PS_5_Cl, and all peaks were well matched with the argyrodite structure. The argyrodite diffraction peak at 30–32° shows a higher angle shift, and the co-doped electrolyte has a higher angle shift than the single-doped electrolytes. This is because the substitution of Al^3+^ and Cl^−^ with smaller ionic radii at Li^+^ and S^2−^, respectively, affected the reduction in the lattice parameters of the argyrodite phase. Therefore, it was estimated that the XRD peak shift occurred as a result of the strain effect on the structure with the increase in the substitution amount of Al and Cl. XRD Rietveld refinement was used to reveal the detailed structure and site in the co-doped Li_5.4_Al_0.1_PS_4.7_Cl_1.3_ electrolyte. The fitted pattern and refined lattice parameters are shown in Figure S2 and Table S1. Raman spectroscopy was applied to the electrolyte to perform a microcrystal analysis of doping. Figure 3b shows the results of Raman spectroscopy for solid electrolytes with an argyrodite crystal phase. The peak that occurred near 420 cm^−1^ was PS_4_^3−^, which is the basic unit in the argyrodite structure. Through this, it can be confirmed that Al^3+^ continuously maintained the normal argyrodite structure despite the substitution. The Al-bonding-related peak after Al substitution could not be confirmed. From this, it was considered that a small amount of Al was substituted in the solid electrolyte structure, which could not be detected through Raman spectroscopy. 

XPS analysis was also performed to analyze the interfacial reaction of the solid electrolyte. XPS P 2p, S 2p, Cl 2p, and Al 2p spectra of solid electrolytes are shown in Figure 3c–f, and their survey and Li 1s spectra are shown in Figure S3a,b. Figure 3c shows the P 2p spectrum. Double peaks appeared at 133.59 eV and 132.08 eV, corresponding to PS_4_^3−^ [33,34]. The assignment of two different binding energies to PS_4_^3−^ reflects that this anion was located in an environment with different crystallinity or has structural disorder, as amorphization often shifts the binding energy in the XPS spectrum [35,36]. The peak located at high binding energy (133.59 eV) was very weak, which could be related to the P-S-P bridging sulfur structure [34,37]. Interestingly, it was observed that the high-binding-energy (133.59 eV) peak decreased and the low-binding-energy (132.08 eV) peak increased in the doped solid electrolyte, which means that the region corresponding to P_2_S_5_ became smaller. The relative areas corresponding to high and low binding energies were 69.1% and 31.0% in the (x = 0, y = 0) electrolyte, 55.4% and 44.6% in the (x = 0.5, y = 0) electrolyte, 35.2% and 64.8% in the (x = 0, y = 0.15) electrolyte, and 41.6% and 58.4% in the (x = 0.3, y = 0.1) electrolyte, respectively. Figure 3d shows the S 2p spectrum. Large peaks appeared at approximately 163.74 eV and 161.74 eV, corresponding to PS_4_^3−^, and small peaks appeared at 166.77 eV and 164.83 eV, corresponding to P_2_S_5_ [38]. The relative areas corresponding to PS_4_^3−^ and P_2_S_5_ were 80.2% and 19.8% in the (x = 0, y = 0) electrolyte and 74.5% and 25.5% in the (x = 0.5, y = 0) electrolyte. The decrease in the relative area of PS_4_^3−^ may be due to the disorder in the increased Cl^−^/S^2−^ sites owing to Cl doping and the sharing of Cl^−^ and S^2−^ in the structure. PS_4_^3−^ and P_2_S_5_ were 83.6% and 16.4% in the (x = 0, y = 0.15) electrolyte, and they were 90.4% and 9.6% in the (x = 0.3, y = 0.1) electrolyte. It can be seen that the relative area corresponding to PS_4_^3−^ greatly increased. Figure 3e shows the Cl 2p spectrum. All electrolytes showed doublets (200.58 eV and 198.91 eV) for the Cl^−^ ions of argyrodite. The doping of Cl does not appear to affect the change in the doublet. Figure 3f shows the Al 2p spectrum. In the case of the (x = 0, y = 0) and (x = 0.5, y = 0) electrolytes without Al substitution, only the noise signal was recorded, and for (x = 0, y = 0.15) and (x = 0.3, y = 0.1) electrolytes, a new peak corresponding to Al binding (74.5 eV) appeared. The splitting of the Al 2p doublet is lower than the resolution of the Al Kα X-ray source. Thus, it is not split into Al 2p_1/2_ and Al 2p_3/2_ peaks.

### 3.4. Electrochemical Properties of Co-Doped Solid Electrolyte

**Figure 4:**
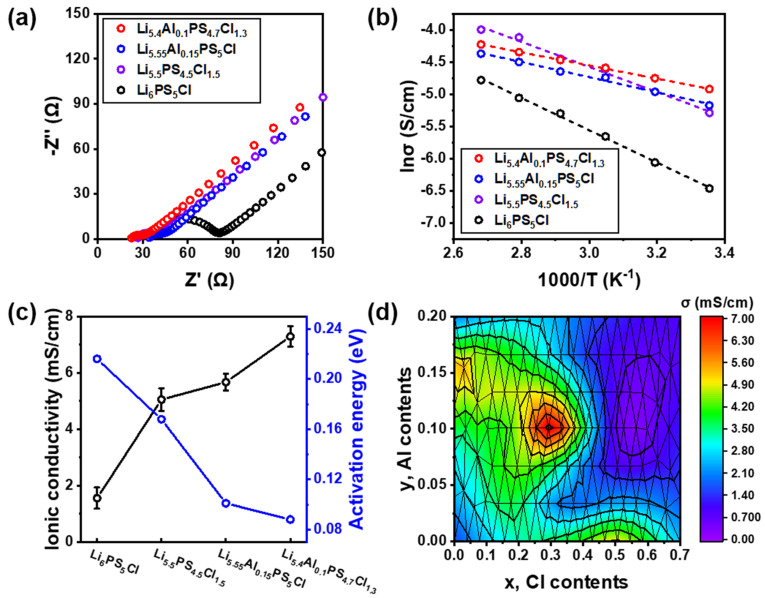
(**a**) Nyquist plots of Li_6_PS_5_Cl, Li_5.5_PS_4.5_Cl_1.5_, Li_5.55_Al_0.15_PS_5_Cl, and Li_5.4_Al_0.1_PS_4.7_Cl_1.3_ electrolytes measured at 25 °C. (**b**) Arrhenius plots of Li_6_PS_5_Cl, Li_5.5_PS_4.5_Cl_1.5_, Li_5.55_Al_0.15_PS_5_Cl, and Li_5.4_Al_0.1_PS_4.7_Cl_1.3_ electrolytes measured in the range of 25 to 100 °C. (**c**) E_a_ and ionic conductivity of Li_6_PS_5_Cl, Li_5.5_PS_4.5_Cl_1.5_, Li_5.55_Al_0.15_PS_5_Cl, and Li_5.4_Al_0.1_PS_4.7_Cl_1.3_ electrolytes. (**d**) Ionic conductivity of Li_6−x−3y_Al_y_PS_5−x_Cl_1+x_ (0 ≤ x ≤ 0.2, 0 ≤ y ≤ 0.7) electrolytes as a function of Cl and Al contents.

Solid electrolytes with the highest ionic conductivities after single doping (x = 0.5, y = 0; x = 0, y = 0.15) and double doping (x = 0.3, y = 0.1) were used for the analysis of electrochemical properties. Nyquist plots measured at 25 °C are shown in Figure 4a and Figure S4. Figure S4a shows a schematic diagram of the cell geometry for the impedance measurement. The EIS data in Figure S4c–f were modeled using the equivalent circuit in Figure S4b. Resistor (R) is the bulk ionic resistance, grain boundary resistance, and charge-transfer resistance between the solid electrolyte and the electrodes. R_s_ is the ionic resistance and R_ct_ is the charge transfer resistance, which is the resistance of the electrolyte. The constant-phase element (CPE, Q) is used for the non-ideal capacitance that can occur due to the non-uniformity and porosity of the electrochemical material and interface, and it accounts for the infinitely diffusing Warburg element. Warburg (Z_W_) is used for resistance caused by mass ions (diffusion), which are the most prominent at low frequencies. The measured R_ct_ values of Li_6_PS_5_Cl, Li_5.5_PS_4.5_Cl_1.5_, Li_5.55_Al_1.5_PS_5_Cl, and Li_5.4_Al_0.1_PS_4.7_Cl_1.3_ electrolytes were 53 Ω, 13.87 Ω, 12.36 Ω, and 9.6 Ω, respectively. The R_ct_ of the co-doped electrolyte was smaller than that of the single-doped electrolytes. Such a low resistance ensures the excellent ionic conductivity performance of the solid electrolyte. The Arrhenius plots, calculated by measuring the ionic conductivity at a temperature range of 25–100 °C (Figure S5), are shown in Figure 4b. The slope of the co-doped electrolyte is smaller than that of the single-doped electrolytes. The E_a_ and ionic conductivity of the solid electrolyte are shown in Figure 4c. When the Cl and Al contents were x = 0.3 and y = 0.1, respectively, the ionic conductivity was the highest. The Li_5.4_Al_0.1_PS_4.7_Cl_1.3_ electrolyte obtained an ionic conductivity of 7.29 × 10^−3^ S cm^−1^ and an E_a_ of 0.09 eV, and the ionic conductivity was 4.7 times higher as compared to that of Li_6_PS_5_Cl. In the Li_6_PS_5_Cl structure, the substitution of the halogen Cl^−^ ion increases the disorder of the Cl^−^/S^2−^ site to increase the Li^+^ site vacancy concentration [30], and the substitution of the Al^3+^ cation, having a radius similar to that of the Li^+^ ion, also increases the Li^+^ site vacancy concentration [24]. Due to the generated vacancy site, a diffusion path is formed, and Li^+^ ions move. This causes the ionic conductivity to increase by several times compared to the parent phase. The two effects work together to lower the activation energy and increase the ionic conductivity and Li diffusion, provided that the solubility limit of the lattice for either dopant is not exceeded. This increases by a lot more than conducting each individually. This indicates that Cl and Al doping can simultaneously improve the ionic conductivity while decreasing E_a_. Figure 4d and Table 1 show the ionic conductivities of Li_6−x−3y_Al_y_PS_5−x_Cl_1+x_ (0 ≤ x ≤ 0.7, 0 ≤ y ≤ 0.2) electrolytes measured at 25 °C. In this study, the solid electrolyte with the composition Li_5.4_Al_0.1_PS_4.7_Cl_1.3_ showed the best ionic conductivity.

## 4. Conclusions

In this study, we have reported an argyrodite solid electrolyte, Li_6_PS_5_Cl, for all-solid-state lithium batteries with improved ionic conductivity via the co-doping of Cl and Al elements. The argyrodite structure of Li_6_PS_5_Cl was maintained after Cl and Al doping, and the proper concentration of doping elements increased the purity of the argyrodite structure. The electrochemical analysis revealed that the substitution of cations and anions greatly improved the ionic conductivity of solid electrolytes due to the increase in vacancies, which provided pathways for Li^+^ diffusion. The prepared Li_5.4_Al_0.1_PS_4.7_Cl_1.3_ electrolyte showed the highest ionic conductivity of 7.29 × 10^−3^ S cm^−1^ at room temperature, which was more than 4.7 times that of Li_6_PS_5_Cl. The Arrhenius plot further elucidated a low E_a_ of 0.09 eV for the Li_5.4_Al_0.1_PS_4.7_Cl_1.3_ electrolyte. This study provides an effective approach to synthesizing efficient solid electrolytes for all-solid-state batteries and may contribute to further studies related to other battery systems.

## Figures and Tables

**Table 1:** Ionic conductivity of Li_6−x−3y_Al_y_PS_5−x_Cl_1+x_ (0 ≤ x ≤ 0.2, 0 ≤ y ≤ 0.7) electrolytes as a function of Cl and Al contents.

Ionic Conductivity (mS/cm)		x, Cl Contents					
		0	0.2	0.3	0.4	0.5	0.7
**y, Al contents**	**0**	1.56	-	3.6	-	5.05	2.57
	**0.05**	2.62	4.21	2.16	1.34	-	-
	**0.1**	3.52	4.6	7.29	3.83	0.64	1.24
	**0.15**	5.67	-	3.36	1.71	0.80	-
	**0.2**	2.00	-	2.49	-	1.32	0.88

## Data Availability

The data are contained within the article.

## Supplementary Materials

The following supporting information can be downloaded at: https://www.mdpi.com/article/10.3390/nano12244355/s1, Figure S1: FE-SEM images of Li_5.4_Al_0.1_PS_4.7_Cl_1.3_ electrolytes. Figure S2: XRD pattern of and the corresponding Rietveld refinement of Li_5.4_Al_0.1_PS_4.7_Cl_1.3_ electrolyte. Figure S3: XPS spectra of Li_6_PS_5_Cl, Li_5.5_PS_4.5_Cl_1.5_, Li_5.55_Al_0.15_PS_5_Cl, and Li_5.4_Al_0.1_PS_4.7_Cl_1.3_ electrolytes for (a) Survey, and (b) Li 1s. Figure S4: (a) Schematic diagram of cell geometry for impedance measurement. (b) Equivalent circuit for modeling the EIS data. Nyquist plots of (c) Li_6_PS_5_Cl, (d) Li_5.5_PS_4.5_Cl_1.5_, (e) Li_5.55_Al_0.15_PS_5_Cl, and (f) Li_5.4_Al_0.1_PS_4.7_Cl_1.3_ electrolytes, respectively. Experimental data (circles) was fitted with the simulated data from equivalent circuit modeling (lines). Figure S5: Nyquist plots of the (a) Li_6_PS_5_Cl, (b) Li_5.5_PS_4.5_Cl_1.5_, (c) Li_5.55_Al_0.15_PS_5_Cl, and (d) Li_5.4_Al_0.1_PS_4.7_Cl_1.3_ electrolytes measured from 25 to 100 °C, respectively.; Table S1: Rietveld refinements data of Li_5.4_Al_0.1_PS_4.7_Cl_1.3_ electrolyte.
