# Supplementary material for: Cl- and Al-Doped Argyrodite Solid Electrolyte Li6PS5Cl for All-Solid-State Lithium Batteries with Improved Ionic Conductivity
Source: Nanomaterials (Basel). 2022 Dec 7;12(24):4355. doi: 10.3390/nano12244355 (PMC9783369; doi:10.3390/nano12244355)
Supplement: Supplementary file 1 [file nanomaterials-12-04355-s001.zip › nanomaterials-1950472-supplementary.pdf]

nanomaterials

Supplementary Information

Cl- and Al-Doped Argyrodite Solid Electrolyte Li_6PS_5Cl for All-Solid-State Lithium Batteries with Improved Ionic Conductivity

Yeong Jun Choi [1,2], Sun-I Kim [1], Mingyu Son [1,2], Jung Woo Lee [2,*] and Duck Hyun Lee [1,3,*]

1 Green Materials and Processes R&D Group, Korea Institute of Industrial Technology, Ulsan 44413, Republic of Korea; yjchoi@kitech.re.kr (Y.J.C.); sunikim@kitech.re.kr (S.-I.K.); smg0616@kitech.re.kr (M.S.)
2 Department of Materials Science and Engineering, Pusan National University, Busan 46241, Republic of Korea
3 School of Materials Science and Engineering, Andong National University, Andong 36729, Republic of Korea
* Correspondence: jungwoolee@pusan.ac.kr (J.W.L.); dulee@kitech.re.kr (D.H.L.)

Citation: Choi, Y.J.; Kim, S.-I.; Son, M.; Lee, J.W.; Lee, D.H. Cl- and Al-Doped Argyrodite Solid Electrolyte Li_6PS_5Cl for All-Solid-State Lithium Batteries with Improved Ionic Conductivity. *Nanomaterials* **2022**, *12*, x. https://doi.org/10.3390/xxxxx

Academic Editors: Dawei Su, Jun Liu

Received: 19 September 2022
Accepted: 5 December 2022
Published: 7 December 2022

Publisher's Note: MDPI stays neutral with regard to jurisdictional claims in published maps and institutional affiliations.

Copyright: © 2022 by the authors. Licensee MDPI, Basel, Switzerland. This article is an open access article distributed under the terms and conditions of the Creative Commons Attribution (CC BY) license (https://creativecommons.org/licenses/by/4.0/).

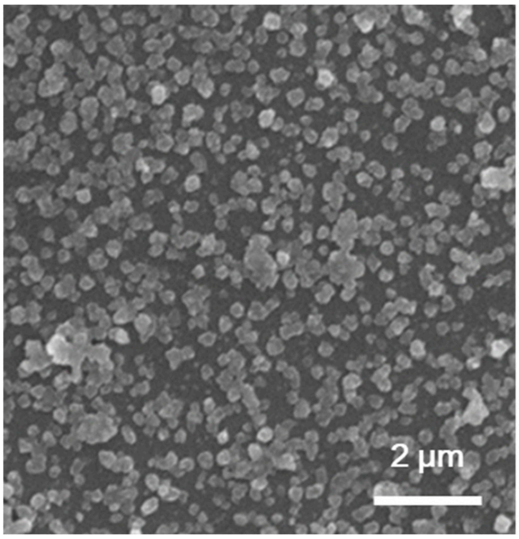

Figure S1. FE-SEM images of $Li_{5.4}Al_{0.1}PS_{4.7}Cl_{1.3}$ electrolytes.

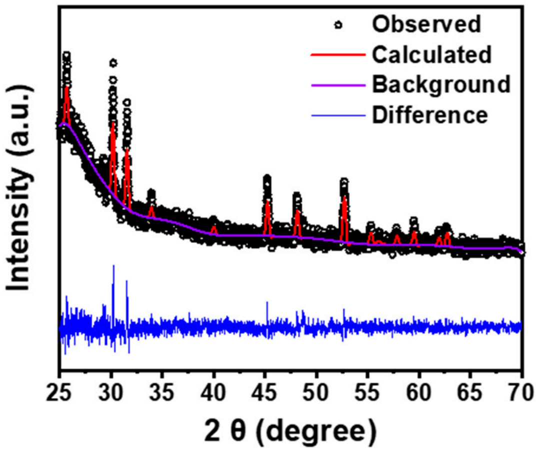

Figure S2. XRD pattern of and the corresponding Rietveld refinement of $Li_{5.4}Al_{0.1}PS_{4.7}Cl_{1.3}$ electrolyte.

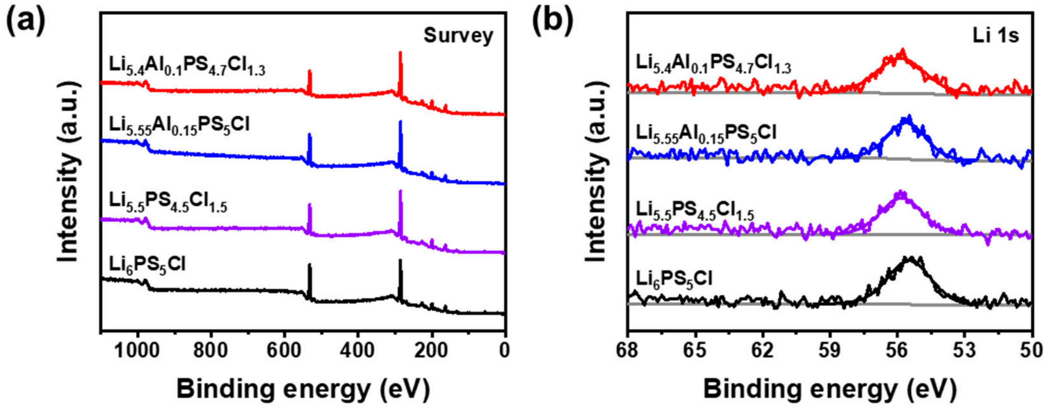

Figure S3. XPS spectra of Li_6PS_5Cl, $Li_{5.5}PS_{4.5}Cl_{1.5}$, $Li_{5.55}Al_{0.15}PS_5Cl$, and $Li_{5.4}Al_{0.1}PS_{4.7}Cl_{1.3}$ electrolytes for **(a)** Survey, and **(b)** Li 1s.

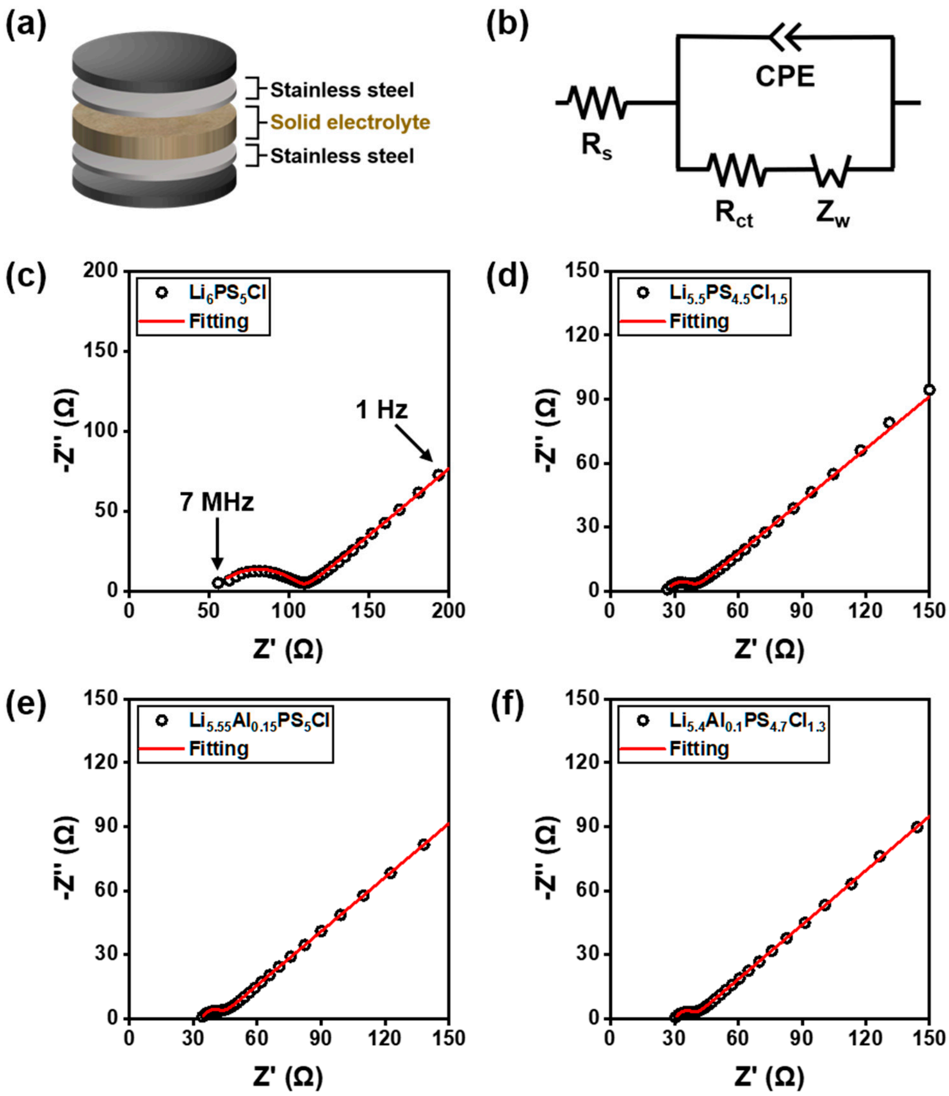

Figure S4. (a) Schematic diagram of cell geometry for impedance measurement. **(b)** Equivalent circuit for modeling the EIS data. Nyquist plots of **(c)** Li_6PS_5Cl, **(d)** $Li_{5.5}PS_{4.5}Cl_{1.5}$, **(e)** $Li_{5.55}Al_{0.15}PS_5Cl$, and **(f)** $Li_{5.4}Al_{0.1}PS_{4.7}Cl_{1.3}$ electrolytes, respectively. Experimental data (circles) was fitted with the simulated data from equivalent circuit modeling (lines).

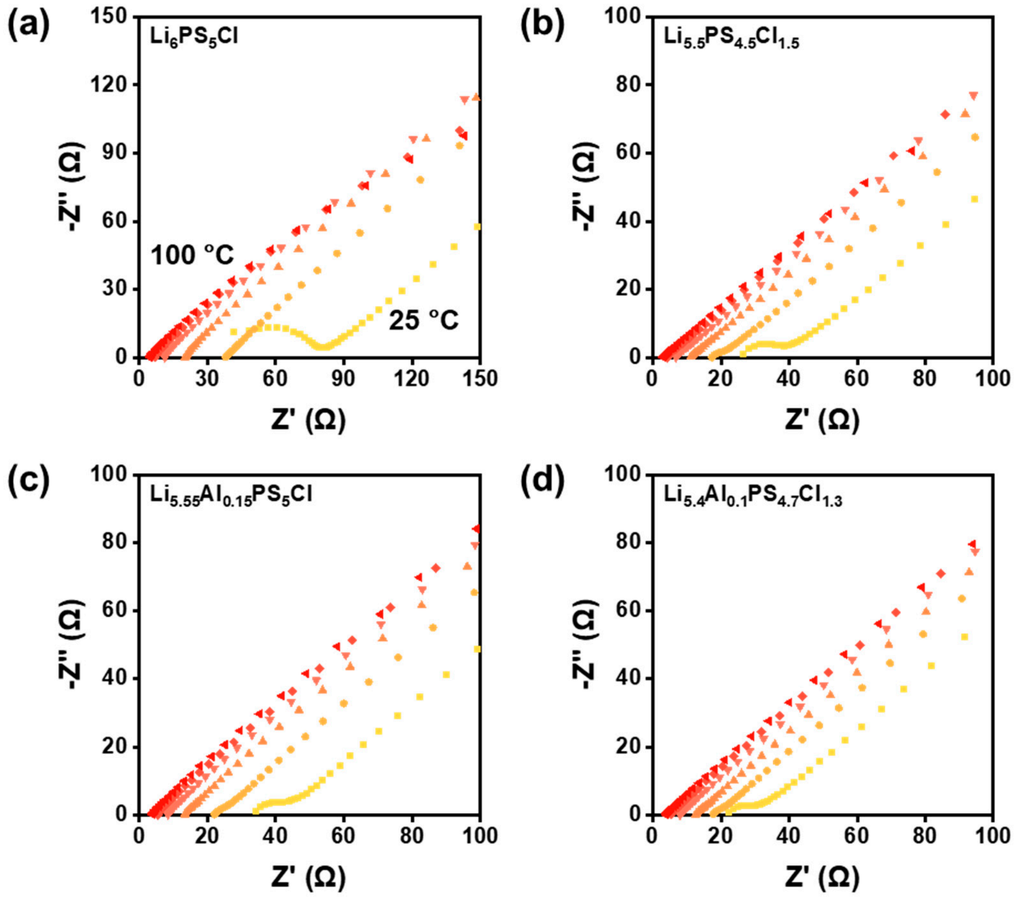

Figure S5. Nyquist plots of the **(a)** Li_6PS_5Cl, **(b)** $Li_{5.5}PS_{4.5}Cl_{1.5}$, **(c)** $Li_{5.55}Al_{0.15}PS_5Cl$, and **(d)** $Li_{5.4}Al_{0.1}PS_{4.7}Cl_{1.3}$ electrolytes measured from 25 to 100 °C, respectively.

Table S1. Rietveld refinements data of $Li_{5.4}Al_{0.1}PS_{4.7}Cl_{1.3}$ electrolyte.

space group $F\bar{4}3m$, a=10.28 Å, R_{wp}=19.51 %						
**atom**	**site**	**x**	**y**	**z**	**OCC.**	**Uiso (Å^2)**
Li1	24g	0.25	0.25	0.36369	1	-2.62899
P1	4b	0.5	0	0	1	0.05134
S1	4a	0.25	0.25	0.25	1	-1.02402
S2	4c	0.12684	0.37316	0.87316	1	-0.05982
Cl1	4a	0	0	0	1	-0.11452
